# Nasopharyngeal Microbiome Diversity Changes over Time in Children with Asthma

**DOI:** 10.1371/journal.pone.0170543

**Published:** 2017-01-20

**Authors:** Marcos Pérez-Losada, Lamia Alamri, Keith A. Crandall, Robert J. Freishtat

**Affiliations:** 1 Computational Biology Institute, George Washington University, Ashburn, Virginia, United States of America; 2 Division of Emergency Medicine, Children’s National Medical Center, Washington DC, United States of America; 3 CIBIO-InBIO, Centro de Investigação em Biodiversidade e Recursos Genéticos, Universidade do Porto, Campus Agrário de Vairão, Vairão, Portugal; Columbia University, UNITED STATES

## Abstract

**Background:**

The nasopharynx is a reservoir for pathogens associated with respiratory illnesses such as asthma. Next-generation sequencing (NGS) has been used to characterize the nasopharyngeal microbiome of infants and adults during health and disease; less is known, however, about the composition and temporal dynamics (i.e., longitudinal variation) of microbiotas from children and adolescents. Here we use NGS technology to characterize the nasopharyngeal microbiomes of asthmatic children and adolescents (6 to 18 years) and determine their stability over time.

**Methods:**

Two nasopharyngeal washes collected 5.5 to 6.5 months apart were taken from 40 children and adolescents with asthma living in the Washington D.C. area. Sequence data from the 16S-V4 rRNA gene region (~250 bp) were collected from the samples using the MiSeq platform. Raw data were processed in mothur (SILVA123 reference database) and Operational Taxonomic Units (OTU)-based alpha- and beta-diversity metrics were estimated. Relatedness among samples was assessed using PCoA ordination and Procrustes analyses. Differences in microbial diversity and taxon mean relative proportions were assessed using linear mixed effects models. Core microbiome analyses were also performed to identify stable and consistent microbes of the nasopharynx.

**Results and Discussion:**

A total of 2,096,584 clean 16S sequences corresponding to an average of 167 OTUs per sample were generated. Representatives of Moraxella*, Staphylococcus*, *Dolosigranulum*, *Corynebacterium*, *Prevotella*, Streptococcus*, Haemophilus*, Fusobacterium* and a Neisseriaceae genus accounted for 86% of the total reads. These nine genera have been previously found in the nasopharynxes of both infants and adults, but in different proportions. OTUs from the five genera highlighted (*) above defined the nasopharyngeal core microbiome at the 95% level. No significant differences in alpha- and beta-diversity were observed between seasons, but bacterial mean relative proportions of *Haemophilus*, *Moraxella*, *Staphylococcus* and *Corynebacterium* varied significantly between summer-fall and age groups (inter-patient variation). Additionally, OTUs varied significantly within patients between time points in 35 of the 40 patients analyzed. Future cross-sectional studies should be mindful of the temporal dynamics of the nasopharyngeal microbiota.

## Introduction

The application of next-generation sequencing (NGS) technology to microbial communities residing in the respiratory airways (i.e., airway microbiome) has shown that bacteria may play a significant role in the onset, development, and severity of respiratory diseases such as asthma [1–4]. Both metataxonomics and metagenomics (see [5] for distinction) have demonstrated that airway microbiome composition and structure vary between healthy and diseased individuals and revealed significant associations between representatives of several opportunistic pathogens (*Moraxella*, *Streptococcus*, *Haemophilus*, *Neisseria* and *Staphylococcus*), clinical conditions, and stages of asthma [3, 6–12]. Moreover, microbiome research has shown that all of these pathogens are normal and transient residents of the nasopharynx [10]. As such, they are mainly asymptomatic, but through the nasopharynx they can directly spread to other sections of the respiratory tract and potentially cause asthma, otitis media or pneumonia; or invade the bloodstream to cause sepsis and meningitis [8, 13–15].

Given the general importance of the nasopharynx as a reservoir for microbes associated with respiratory infections [8, 16, 17], several NGS studies have investigated the microbial composition of the nasopharynx during health and disease in both infants and adults, and in relation to clinical factors. Most of these microbiome studies, however, have been designed in a cross-sectional setting (i.e., one time point) [14, 18–24]. Less is known about the temporal dynamics of the nasopharyngeal microbiota (i.e., microbial succession) within patients and, therefore, the influence of changing environmental factors (e.g., seasonality) on the structuring of microbial communities over time. Moreover, temporal variation complicates the prediction of microbial profiles associated with disease progression, the assessment of intra- and inter-patient variation, and the study of microbe-host interactions. Gut microbiome research is increasingly showing that longitudinal (temporal) changes in bacterial community composition and function are associated with disease [25–27]. Similarly, longitudinal studies of the nasopharyngeal microbiome in infants have revealed that certain microbial groups change over the first two years of life in both healthy and asthmatic children [8, 15] and that microbiota profiles vary strongly with the seasons [8, 10]. Additionally, these studies have also shown a high inter-patient microbial diversity, so no nasopharyngeal core microbiome could be defined at the OTU level.

Here, we use NGS technology to explore the nasopharyngeal microbiome. We couple targeted 16S rRNA sequencing (metataxonomics) with a sophisticated analytical pipeline to characterize the nasal microbiome diversity of asthmatic children and adolescents (ages 6 to 18 years) and determine its stability (i.e., microbial succession) over time (5.5 to 6.5 months) and across seasons. Our results complement previous microbiome studies of the nasopharynx focused on younger children (<2 years of age) and adults.

## Materials and Methods

### Ethics

All participants in this study were part of the AsthMaP2 (Asthma Severity Modifying Polymorphisms) Study. AsthMaP2 is an ongoing study of urban children and adolescents designed to find associations among airway microbes, environmental exposures, allergic sensitivities, genetics, and asthma. AsthMaP2 and the study presented here were approved by the Children's National Medical Center Institutional Review Board (Children's National IRB), which requires that consent is obtained and documented prior to conducting study procedures and collection of samples for research. Written consent was obtained from all independent participants or their legal guardians using the Children's National IRB approved informed consent documents (IRB No PRO00002517).

### Samples and molecular analyses

Two nasal washes (W1 and W2) were collected from 40 children and adolescents (ages 6 to 18 years; mean = 11 years) enrolled in the AsthMaP2 study in two consecutive visits (5.5 to 6.5 months apart) at Children’s National Medical Center (Washington, DC) [28]. Children were recruited from the metropolitan Washington, DC, area; they had been physician-diagnosed with asthma for at least one year prior to recruitment and have been followed up for another year afterwards. Individuals who reported a medical history of chronic or complex cardiorespiratory disease were ineligible.

Washes were procured by instilling 5 ml of isotonic sterile saline buffer into each nare, holding it for 10 seconds and then blowing into a specimen collection container. Total DNA was extracted using the QIAGEN QIAamp DNA Kit (Catalog # 51304). Before adding the ATL buffer, samples were pre-incubated in 100 uL of lysozyme-TE buffer pH = 8.0 for 30 minutes at 37°C. All extractions yielding >50 ng of total DNA (as indicated by NanoDrop 2000 UV-Vis Spectrophotometer measuring) were further processed. DNA extractions were prepared for sequencing using the Schloss’ MiSeq_WetLab_SOP protocol (09.2015) in Kozich et al. [29]. Each DNA sample was amplified for the V4 region (~250 bp) of the 16S rRNA gene and libraries were sequenced in a single run of the Illumina MiSeq sequencing platform at University of Michigan Medical School. Sequence data have been deposited in GenBank under SRA accession number SRP069020.

### Analyses

Raw FASTQ files were processed in mothur v1.35.1 [30]. Default settings were used to minimize sequencing errors as described in Schloss et al. [31]. Clean sequences were aligned to the SILVA123-based bacterial reference alignment at http://www.mothur.org. Chimeras were removed using uchime [32] and non-chimeric sequences were classified using the naïve Bayesian classifier of Wang et al. [33]. Sequences were clustered into Operational Taxonomic Units (OTUs) at the 0.03 threshold (species level). OTU sequence representatives and taxonomy were imported (BIOM format) into QIIME [34] for subsequent analyses. The mothur OTU table was filtered to a minimum of 2 observations (sequences) per OTU. Samples were subsampled (rarefaction analysis) to the smallest sample size (2,288 sequences) to remove the effect of sample size bias on community composition.

Trees for phylogenetic diversity calculations were constructed using FastTree [35]. Taxonomic alpha-diversity was estimated as the number of observed OTUs, but also by the Good’s coverage, Chao1, and Shannon indices. Phylogenetic alpha-diversity was calculated by the Faith’s phylogenetic diversity index [36]. Phylogenetic (unweighted and weighted unifrac) beta-diversity metrics were calculated between pairs of samples. Dissimilarity between samples was estimated using principal coordinates analysis (PCoA) and both unifrac distances. Procrustes analysis comparing PCoA plots (weighted unifrac distances) of W1 and W2 groups [37] was performed with 10,000 Monte Carlo iterations. Linear mixed effects models (LME) analysis was applied to both alpha-diversity indices and taxa mean relative proportions (response), while accounting for non-independence of intra-patient samples (random effect) and seasons, age (years), gender, body mass index (BMI), National Asthma Education and Prevention Program (NAEPP) severity level [38], and ethnic background (predictors) (S1 Table). Phylogenetic distances were also compared between seasons using the non-parametric adonis test from the vegan R’s library [39] while accounting for random effects and all predictors above. Significance was determined through permutation testing after 10,000 permutations. Finally, differences in genus mean relative proportions between visits (W1 *versus* W2) were assessed for each patient using the Fisher’s exact test. The nasopharyngeal core microbiome (OTUs that are present in a certain percentage of samples) in our 80 samples was identified at different levels of stringency ranging from 50% to 100% of the samples. Bonferroni or Benjamini-Hochberg FDR multiple test correction methods were applied. All analyses were performed in mothur, QIIME, STAMP [40], and RStudio [41].

## Results and Discussion

### The nasopharyngeal microbiome of asthmatic children

**Fig 1:**
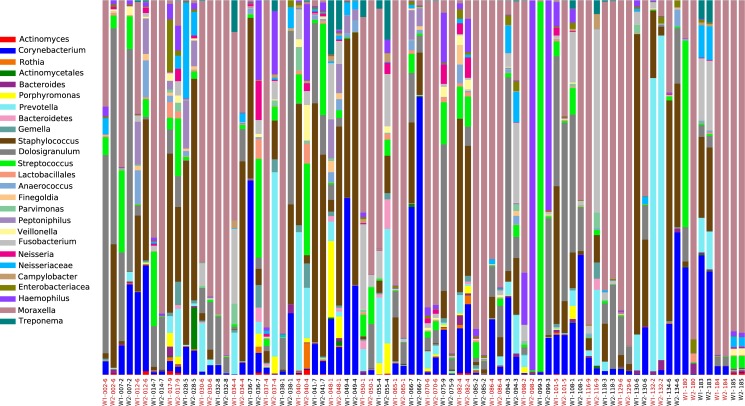
Taxonomic profiles of 80 nasopharyngeal microbiomes from 40 asthmatic children. Only OTUs with a minimum total observation count of 0.1% are shown. W1 = nasal microbiome from wash 1; W2 = nasal microbiome from wash 2 collected 5.5 to 6.5 months later. Sample pairs are alternatively colored in red and black to facilitate their visualization.

A total of 80 nasal microbiomes corresponding to 40 asthmatic children (two samples per patient 5.5 to 6.5 months apart) were analyzed via MiSeq sequencing of 16S rRNA V4 amplicons. A total of 2,096,584 sequences ranging from 2,288 to 60,806 sequences per sample (mean = 26,207.3; median = 22,381.5) were obtained, after quality control analyses and OTU filtering. From these data, we identified 34–394 OTUs (mean = 167) per sample belonging to 397 classified bacterial genera and 23 classified phyla (S2 and S3 Tables). About 86% of the sequences corresponded to the following nine genera: *Moraxella* (35.3%), *Staphylococcus* (13.9%), *Dolosigranulum* (9.3%), *Corynebacterium* (8.8%), *Prevotella* (6.0%), *Streptococcus* (4.9%), *Haemophilus* (3.5%), *Fusobacterium* (3.0%) and Neisseriaceae genus (1.3%) (see Fig 1 and S3 Table).

Our study does not include samples from healthy children, hence we cannot assess to what extent the microbial profiles we observe here are unique to asthmatic children. Moreover, we are not aware of any study that has described the nasopharyngeal microbiome of healthy children (2 to 18 years old). For comparative purposes, we have then compiled results from several published studies describing the nasopharyngeal microbiomes of non-asthmatic infants (defined as children between the ages of 1–18 months) and adults (Table 1). Some genera in our study show different mean relative proportions than those seen both in infants (*Corynebacterium*, *Dolosigranulum*, *Flavobacterium*, *Haemophilus*, *Prevotella* and *Streptococcus*) and adults (*Corynebacterium*, *Moraxella*, *Prevotella* and *Streptococcus*), while others seem to show similar mean relative proportions across all age groups (*Fusobacterium*, *Neisseria* and *Staphylococcus*). This comparison, therefore, suggests that the nasopharyngeal microbiome profiles observed here are different from those seen in healthy infants and adults.

**Table 1:** Summary of studies of the nasopharyngeal microbiome in non-asthmatic patients. Number of samples (Ns), age group by month (m) and year (yr), sequenced 16S region, patient condition and genus mean relative proportions (with SD estimates for our study) are indicated. Studies (included ours) are ordered by patient age group.

Study	Teo et al. (2015)	Bogaert et al. (2011)	This study	Hilty et al. (2010)
Ns	487	96	80	40
Age group	Infants (1–12 m)	Infants (18 m)	Children (6–17 yr)	Adults (≥18 yr)
16S	V4	V5–V6	V4	V3-V6
Patient condition	Healthy	Healthy	Asthmatic	Healthy
*Corynebacterium*	10–22	2	8.8 (13.1)	32.8
*Dolosigranulum*	14–26	5	9.3 (13.6)	9.9
*Flavobacterium*	NR	10	<1	<1
*Fusobacterium*	NR	1	3 (6.4)	2.6
*Haemophilus*	NR	20	3.5 (12.6)	4.5
*Moraxella*	9–41	40	35.5 (37.0)	4.8
*Neisseria*	NR	2	<1	<1
*Prevotella*	NR	<1	6 (14.4)	3.5
*Staphylococcus*	11–41	<1	13.9 (18.3)	9.1
*Streptococcus*	NR	12	4.9 (12.8)	9.1

NR = Proportion not reported

The nasopharynx is considered a reservoir for commensal and potentially pathogenic microbes inhabiting the respiratory airways and associated with acute respiratory infections, including asthma [4, 8–10, 16, 42]. All dominant genera reported here for the nasopharynx [several of them including pathogenic species (e.g., *Moraxella*, *Staphylococcus* and *Streptococcus*)] have been detected in other microbiome studies of lower and upper (excluding the nasopharynx) respiratory airways of asthmatics [1, 16, 43, 44]. Thus, our results confirm in children the role of the nasopharynx as a source of potential pathogens for other sections of the respiratory tract [4, 16]. Previous studies of the nasopharyngeal microbiome in both infants [8] and adults [16] with respiratory infections showed that their microbiomes were comprised of the same main genera reported here for asthmatic children and adolescents (ages of 6 to 18 years), although their mean relative proportions varied across studies. Nasopharyngeal microbiomes of infants (1 to 14 months) with acute respiratory infections seem to be dominated by *Haemophilus*, *Streptococcus*, and *Moraxella* [8]; while nasopharyngeal microbiomes of adults with asthma seem to be dominated with *Staphylococcus*, *Corynebacterium*, and *Neisseria* [16]. Given that in our study *Moraxella* and *Staphylococcus* are the dominant taxa, this seems to suggest that microbial succession towards more adult-like configurations in the nasopharynx of asthmatics does not end at early childhood (~2 years) [8, 15] and may take longer than in other body compartments such as the gut [45].

### The asthma core microbiome

The differentiation of the core microbiome of a host meta-organism or host microhabitat is a means to differentiate the stable and consistent members and associations from the whole community [46, 47]. The identification of core members of the meta-organism subsequently allows for the differentiation of core pathways and metabolic functions that are provided by the host—microbe interaction [48]. A core microbiome composed of a variable number of OTUs was observed in the studied nasopharyngeal samples. The least stringent definition of the core (presence in at least 50% of the samples) identified 43 OTUs, while the most stringent definition (presence in all sampled children) included only one OTU of the genus *Staphylococcus*. At the 95% level, the nasopharyngeal core microbiome of asthmatic children was represented by five OTUs of the genera *Moraxella*, *Staphylococcus*, *Streptococcus*, *Haemophilus*, and *Fusobacterium*. These five OTUs may represent fingerprints or biological markers (pulmotypes; [17]) of the nasopharyngeal (and potentially the airway) microbiome in asthmatic children. Most studies of the nasopharyngeal microbiome in infants with and without respiratory infections [8, 10, 15, 49] were not able to define a core microbiome at the OTU level due to the high inter-individual variability of their early (and potentially more dynamic) microbiomes. Using a less strict definition of core microbiome (i.e., OTUs present in more than 50% of all samples and representing >0.1% of the sequences), Bogaert et al. [10] described a core microbiome in children 18 months of age composed of *Moraxella*, *Haemophilus*, *Enhydrobacter*, *Streptococcus*, *Dolosigranulum*, and *Corynebacterium*. All these genera were observed in our 50% core microbiome except *Enhydrobacter*, and three genera (*Moraxella*, *Haemophilus*, and *Streptococcus*) were observed in our 95% core microbiome. Therefore, if the consistency and specificity (i.e., dominant in asthmatics and absent in healthy controls) of these taxa across all “ages of asthma” are confirmed, they could potential be used to phenotype this complex and heterogeneous disease (i.e., pulmotypes).

### Temporal diversity in the nasopharyngeal microbiome

**Fig 2:**
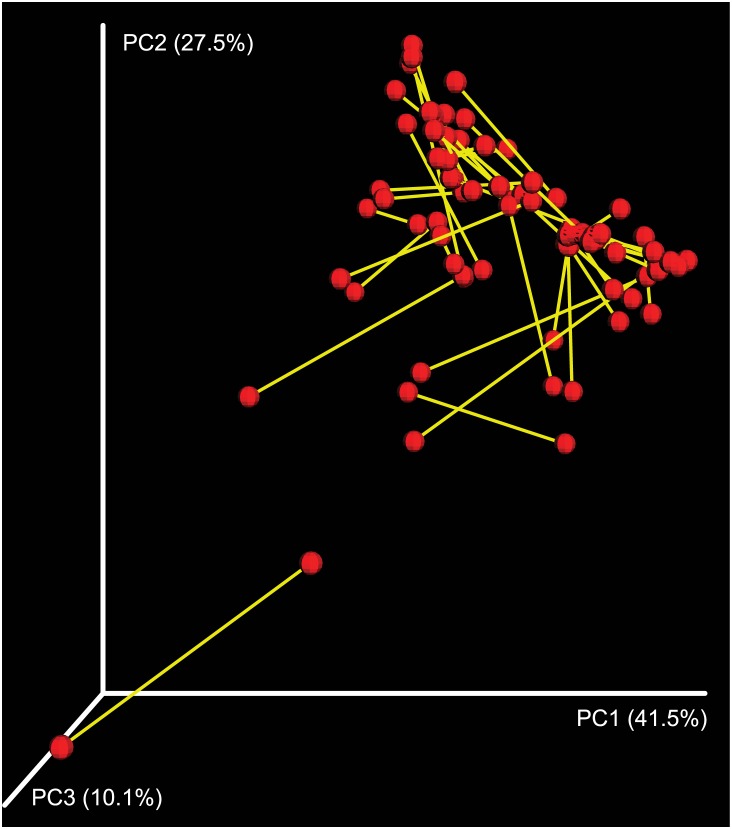
Procrustes analysis of 40 nasopharyngeal sample pairs collected 5.5 to 6.5 months apart. Sample pairs (red dots) collected from the same patient are connected by a yellow bar. The length of the bar is proportional to the dissimilarity between sample pairs.

Procrustes analysis comparing PCoA plots of W1 (nasal wash 1) and W2 (nasal wash 2) microbiomes collected 5.5 to 6.5 months apart showed a great dissimilarity between (M^2^ = 0.663) both datasets (Fig 2). Microbial profiles (Fig 1) and pairwise comparisons of the most abundant genera between W1 and W2 samples (S1 Fig) also varied greatly across patients, with some patients showing increases in bacterial mean relative proportions, some showing the opposite trend, and others showing no visible change. Similarly, some bacterial taxa seemed to fluctuate more than others (e.g., *Moraxella* versus *Streptococcus*) between W1 and W2 samples (S1 Fig). Differences in OTU mean relative proportions between W1 and W2 microbiomes were significant in 35 (88%) of the 40 sample pairs analyzed. The number of significant (P< 0.05) OTUs detected varied from one to 19, as indicated by the Fisher’s exact test (effective size ≥1% for the difference in mean relative proportions). All these results combined indicate that members of the nasopharyngeal bacteriome of asthmatic children change over time (5.5 to 6.5 months) on an individual basis. Our study complements previous longitudinal studies revealing differences in microbial composition between infants 2, 6 and 12 months old at risk of developing respiratory infections and asthma [8]. Future research assessing microbial succession and host-microbe interactions during asthma should account for both intra- and inter-patient microbiome variability in the studied cohorts and temporal dynamics at even shorter time spans than those studied here.

**Fig 3:**
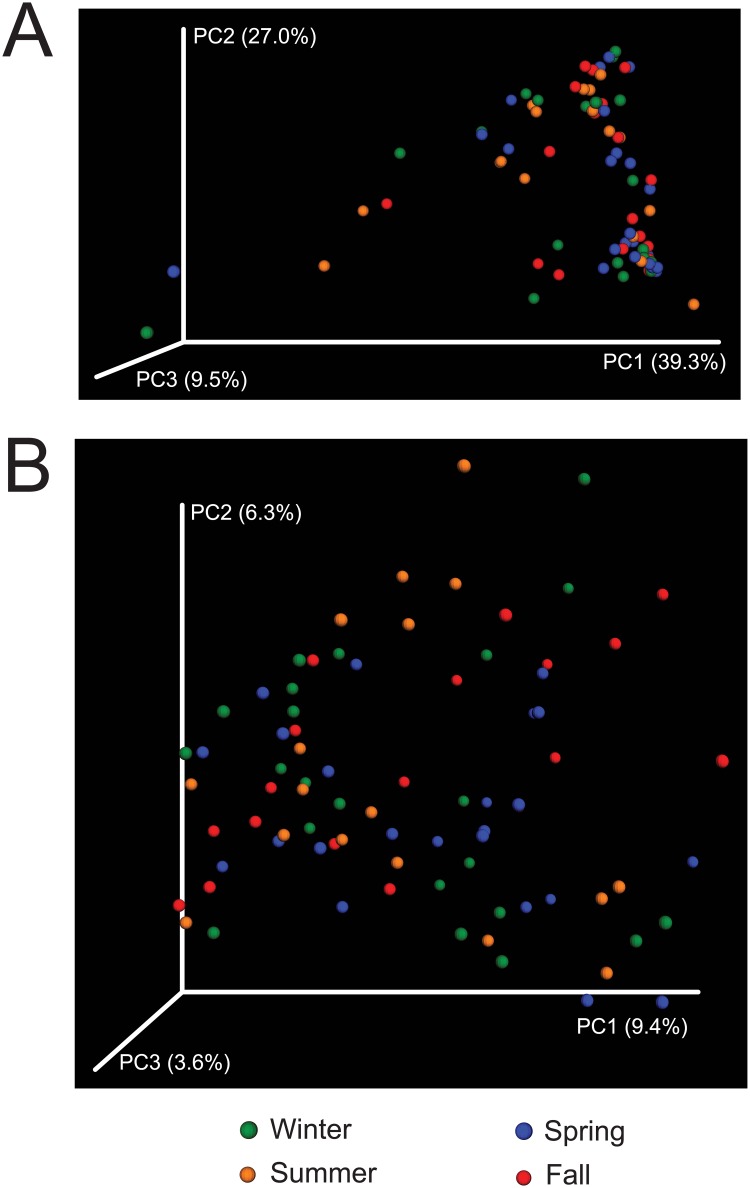
3D Principal Coordinates Analyses of weighted (A) and unweighted (B) unifrac distances between 80 nasopharyngeal microbiomes colored by season.

To further explore inter-patient diversity in our cohort, we investigated if sampling seasonality impacted alpha- and beta-diversity and microbe (genera) mean relative proportions while accounting for non-independence of samples, age (years), gender, BMI, NAEPP severity level, and ethnic background (S1 Table). We did not detect significant differences (P>0.05) in alpha diversity (observed OTUs, Good’s coverage, Chao1, Shannon and Faith–S2 Table) between seasonal groups or any of the confounders in our linear mixed effects models (LME) analyses (data not shown). Additionally, ordination analysis (PCoA—Fig 3) did not reveal clear dissimilarities between nasopharyngeal microbiomes grouped by season for both unifac distances. Similarly, no significant differences (P>0.05) in beta-diversity (unifrac distances) were detected by the adonis tests between seasons while accounting for all other confounders (data not shown). This contrasts with previous nasopharyngeal microbiome studies showing large differences in the microbiota profiles of healthy infants during seasons [10]. No study so far has addressed if those differences are also seen in children and adults.

At the taxon-level, our LME analyses of mean relative proportions of the most abundant genera (Table 2) found no significant differences between seasons for all taxa but *Haemophilus*, which showed a significantly (P = 0.014) higher mean relative proportion in summer (9.3% average) compared to fall (0.3% average) (Table 2). Previous nasopharyngeal microbiome studies have also revealed significant seasonal effects on the proportions of *Haemophilus* and *Moraxella* in infants at risk of developing asthma [8]. Our LME analyses also showed that patient age (6 to 18 years) is significantly associated with variation in the mean relative proportions of *Moraxella*, *Staphylococcus*, and *Corynebacterium*. This agrees with our previous results suggesting that the composition of the nasopharyngeal microbiome changes towards adulthood [8, 15].

**Table 2:** Linear mixed effects (LME) models analysis. Significance (P-values) of the LME analysis of mean relative proportions of most abundant genera (>1% of reads) and clinical and demographic variables. All four seasons were compared, but only the lowest P-values are reported.

	Season	Age	Gender	BMI	Severity Classification	Ethnic Background
*Moraxella*	0.20	**0.002**	0.932	0.139	0.082	0.415
*Staphylococcus*	0.11	**0.045**	0.897	0.578	0.482	0.388
*Dolosigranulum*	0.12	0.610	0.680	0.520	0.270	0.130
*Corynebacterium*	0.12	**0.003**	0.106	0.845	0.335	0.052
*Prevotella*	0.54	0.629	0.769	0.220	0.723	0.055
*Streptococcus*	0.16	0.065	0.456	0.762	0.691	0.387
*Haemophilus*	**0.014**	0.510	0.355	0.594	0.510	0.612
*Fusobacterium*	0.36	0.250	0.220	0.057	0.499	0.846
Neisseriaceae genus	0.35	0.780	0.890	0.330	0.710	0.470

*Haemophilus*: 0.014* (only summer-fall was significant)

Our study (and others) shows that nasopharyngeal microbial communities are not stable; rather they experience structural rearrangements (deterministic or stochastic) over time. Given the exposure of the nasopharynx, such longitudinal changes could be related to environmental factors (e.g., seasonality, diet, pets), although clinical factors (e.g., age, body mass index, neutrophil counts, medication), other microbiotas (i.e., gastrointestinal, oral), anatomical diversity (i.e., nasal microenvironments), and technical biases (e.g., DNA extraction protocol) could also play a role [1–3, 8–10, 42, 50]. Whatever the cause, future cross-sectional studies of the nasopharyngeal microbiome need to be mindful of the short-time dynamics of the microbiotas occupying this human anatomical cavity.

## Supporting Information

S1 Table Clinical and demographic characteristics of the cohort of 40 patients analyzed in this study. (XLSX)

S2 Table Alpha-diversity indices (Good's coverage, observed OTUs, Chao1, Shannon and PD) for 80 nasopharyngeal microbiomes from 40 asthmatic children. (XLSX)

S3 Table Absolute counts and taxonomic classification of OTUs in 80 nasopharyngeal microbiomes from 40 asthmatic children. (XLSX)

S1 Fig Proportions of sequences of the most abundant bacterial genera in 80 nasopharyngeal microbiomes from 40 asthmatic children. (PDF)

## References

[1] Huang YJ, Boushey HA. The microbiome and asthma. Annals of the American Thoracic Society. 2014;11 Suppl 1:S48–51. 24437406 10.1513/AnnalsATS.201306-187MG PMC3972976

[2] Huang YJ, Boushey HA. The microbiome in asthma. The Journal of allergy and clinical immunology. 2015;135(1):25–30. 10.1016/j.jaci.2014.11.011 25567040 PMC4287960

[3] Brar T, Nagaraj S, Mohapatra S. Microbes and asthma: the missing cellular and molecular links. Current opinion in pulmonary medicine. 2012;18(1):14–22. 10.1097/MCP.0b013e32834dccc0 22113000 PMC3532043

[4] Dickson RP, Huffnagle GB. The Lung Microbiome: New Principles for Respiratory Bacteriology in Health and Disease. PLoS pathogens. 2015;11(7):e1004923 10.1371/journal.ppat.1004923 26158874 PMC4497592

[5] Marchesi JR, Ravel J. The vocabulary of microbiome research: a proposal. Microbiome. 2015;3:31 10.1186/s40168-015-0094-5 26229597 PMC4520061

[6] Bisgaard H, Hermansen MN, Bonnelykke K, Stokholm J, Baty F, Skytt NL, et al Association of bacteria and viruses with wheezy episodes in young children: prospective birth cohort study. Bmj. 2010;341:c4978 10.1136/bmj.c4978 20921080 PMC2950260

[7] Castro-Nallar E, Shen Y, Freishtat RJ, Pérez-Losada M, Manimaran S, Liu G, et al Integrating metagenomics and host gene expression to characterize asthma-associated microbial communities. BMC Medical Genomics. 2015;8:50. 26277095 10.1186/s12920-015-0121-1 PMC4537781

[8] Teo SM, Mok D, Pham K, Kusel M, Serralha M, Troy N, et al The infant nasopharyngeal microbiome impacts severity of lower respiratory infection and risk of asthma development. Cell Host & Microbe. 2015;17:704–15. 25865368 10.1016/j.chom.2015.03.008 PMC4433433

[9] Perez-Losada M, Castro-Nallar E, Bendall ML, Freishtat RJ, Crandall KA. Dual Transcriptomic Profiling of Host and Microbiota during Health and Disease in Pediatric Asthma. PloS one. 2015;10(6):e0131819 10.1371/journal.pone.0131819 26125632 PMC4488395

[10] Bogaert D, Keijser B, Huse S, Rossen J, Veenhoven R, van Gils E, et al Variability and diversity of nasopharyngeal microbiota in children: a metagenomic analysis. PloS one. 2011;6(2):e17035 10.1371/journal.pone.0017035 21386965 PMC3046172

[11] Jounio U, Juvonen R, Bloigu A, Silvennoinen-Kassinen S, Kaijalainen T, Kauma H, et al Pneumococcal carriage is more common in asthmatic than in non-asthmatic young men. The clinical respiratory journal. 2010;4(4):222–9. 10.1111/j.1752-699X.2009.00179.x 20887345

[12] Bisgaard H, Hermansen MN, Buchvald F, Loland L, Halkjaer LB, Bonnelykke K, et al Childhood asthma after bacterial colonization of the airway in neonates. The New England journal of medicine. 2007;357(15):1487–95. 10.1056/NEJMoa052632 17928596

[13] Bogaert D, De Groot R, Hermans PW. Streptococcus pneumoniae colonisation: the key to pneumococcal disease. The Lancet Infectious diseases. 2004;4(3):144–54. 10.1016/S1473-3099(04)00938-7 14998500

[14] Garcia-Rodriguez JA, Fresnadillo Martinez MJ. Dynamics of nasopharyngeal colonization by potential respiratory pathogens. The Journal of antimicrobial chemotherapy. 2002;50 Suppl S2:59–73. 10.1093/jac/dkf506 12556435

[15] Biesbroek G, Tsivtsivadze E, Sanders EA, Montijn R, Veenhoven RH, Keijser BJ, et al Early respiratory microbiota composition determines bacterial succession patterns and respiratory health in children. American journal of respiratory and critical care medicine. 2014;190(11):1283–92. 10.1164/rccm.201407-1240OC 25329446

[16] Hilty M, Burke C, Pedro H, Cardenas P, Bush A, Bossley C, et al Disordered microbial communities in asthmatic airways. PloS one. 2010;5(1):e8578 10.1371/journal.pone.0008578 20052417 PMC2798952

[17] Dickson RP, Erb-Downward JR, Huffnagle GB. The role of the bacterial microbiome in lung disease. Expert review of respiratory medicine. 2013;7(3):245–57. 10.1586/ers.13.24 23734647 PMC4007100

[18] Mackenzie GA, Leach AJ, Carapetis JR, Fisher J, Morris PS. Epidemiology of nasopharyngeal carriage of respiratory bacterial pathogens in children and adults: cross-sectional surveys in a population with high rates of pneumococcal disease. BMC infectious diseases. 2010;10:304 10.1186/1471-2334-10-304 20969800 PMC2974682

[19] Feazel LM, Santorico SA, Robertson CE, Bashraheil M, Scott JA, Frank DN, et al Effects of Vaccination with 10-Valent Pneumococcal Non-Typeable Haemophilus influenza Protein D Conjugate Vaccine (PHiD-CV) on the Nasopharyngeal Microbiome of Kenyan Toddlers. PloS one. 2015;10(6):e0128064 10.1371/journal.pone.0128064 26083474 PMC4471099

[20] Prevaes SM, de Winter-de Groot KM, Janssens HM, de Steenhuijsen Piters WA, Tramper-Stranders GA, Wyllie AL, et al Development of the Nasopharyngeal Microbiota in Infants with Cystic Fibrosis. American journal of respiratory and critical care medicine. 2015. 10.1164/rccm.201509-1759OC 26492486

[21] Cremers AJ, Zomer AL, Gritzfeld JF, Ferwerda G, van Hijum SA, Ferreira DM, et al The adult nasopharyngeal microbiome as a determinant of pneumococcal acquisition. Microbiome. 2014;2:44 10.1186/2049-2618-2-44 25671106 PMC4323220

[22] Allen EK, Koeppel AF, Hendley JO, Turner SD, Winther B, Sale MM. Characterization of the nasopharyngeal microbiota in health and during rhinovirus challenge. Microbiome. 2014;2:22 10.1186/2049-2618-2-22 25028608 PMC4098959

[23] Biesbroek G, Bosch AA, Wang X, Keijser BJ, Veenhoven RH, Sanders EA, et al The impact of breastfeeding on nasopharyngeal microbial communities in infants. American journal of respiratory and critical care medicine. 2014;190(3):298–308. 10.1164/rccm.201401-0073OC 24921688

[24] Sakwinska O, Bastic Schmid V, Berger B, Bruttin A, Keitel K, Lepage M, et al Nasopharyngeal microbiota in healthy children and pneumonia patients. Journal of clinical microbiology. 2014;52(5):1590–4. 10.1128/JCM.03280-13 24599973 PMC3993659

[25] Clemente JC, Ursell LK, Parfrey LW, Knight R. The impact of the gut microbiota on human health: an integrative view. Cell. 2012;148(6):1258–70. 10.1016/j.cell.2012.01.035 22424233 PMC5050011

[26] Cho I, Blaser MJ. The human microbiome: at the interface of health and disease. Nature reviews Genetics. 2012;13(4):260–70. 10.1038/nrg3182 22411464 PMC3418802

[27] Caporaso JG, Lauber CL, Costello EK, Berg-Lyons D, Gonzalez A, Stombaugh J, et al Moving pictures of the human microbiome. Genome Biol. 2011;12(5):R50 10.1186/gb-2011-12-5-r50 21624126 PMC3271711

[28] Benton AS, Wang Z, Lerner J, Foerster M, Teach SJ, Freishtat RJ. Overcoming heterogeneity in pediatric asthma: tobacco smoke and asthma characteristics within phenotypic clusters in an African American cohort. The Journal of asthma: official journal of the Association for the Care of Asthma. 2010;47(7):728–34. 20684733 10.3109/02770903.2010.491142 PMC3325290

[29] Kozich JJ, Westcott SL, Baxter NT, Highlander SK, Schloss PD. Development of a dual-index sequencing strategy and curation pipeline for analyzing amplicon sequence data on the MiSeq Illumina sequencing platform. Appl Environ Microbiol. 2013;79(17):5112–20. 10.1128/AEM.01043-13 23793624 PMC3753973

[30] Schloss PD, Westcott SL, Ryabin T, Hall JR, Hartmann M, Hollister EB, et al Introducing mothur: Open-Source, Platform-Independent, Community-Supported Software for Describing and Comparing Microbial Communities. Appl Environ Microb. 2009;75(23):7537–41. 10.1128/AEM.01541-09 PMC2786419 19801464

[31] Schloss PD, Gevers D, Westcott SL. Reducing the effects of PCR amplification and sequencing artifacts on 16S rRNA-based studies. PloS one. 2011;6(12):e27310 10.1371/journal.pone.0027310 22194782 PMC3237409

[32] Edgar RC, Haas BJ, Clemente JC, Quince C, Knight R. UCHIME improves sensitivity and speed of chimera detection. Bioinformatics. 2011;27(16):2194–200. 10.1093/bioinformatics/btr381 21700674 PMC3150044

[33] Wang Q, Garrity GM, Tiedje JM, Cole JR. Naive Bayesian classifier for rapid assignment of rRNA sequences into the new bacterial taxonomy. Appl Environ Microb. 2007;73(16):5261–7. 10.1128/AEM.00062-07 PMC1950982 17586664

[34] Caporaso JG, Kuczynski J, Stombaugh J, Bittinger K, Bushman FD, Costello EK, et al QIIME allows analysis of high-throughput community sequencing data. Nat Methods. 2010;7(5):335–6. 10.1038/nmeth.f.303 20383131 PMC3156573

[35] Price MN, Dehal PS, Arkin AP. FastTree 2-Approximately Maximum-Likelihood Trees for Large Alignments. PloS one. 2010;5(3). 10.1371/journal.pone.0009490 PMC2835736 20224823

[36] Faith DP. Conservation evaluation and phylogenetic diversity. Biol Conserv. 1992;61:1–10.

[37] Gower JC. Generalized Procrustes Analysis. Psychometrika. 1975;40(1):33–51.

[38] Expert Panel Report 3 (EPR-3). Guidelines for the Diagnosis and Management of Asthma—Summary Report 2007. J Allergy Clin Immun. 2007;120(5 Suppl):S94–S138. 17983880 10.1016/j.jaci.2007.09.043

[39] Dixon P. VEGAN, a package of R functions for community ecology. J Veg Sci. 2003;14(6):927–30.

[40] Parks DH, Tyson GW, Hugenholtz P, Beiko RG. STAMP: statistical analysis of taxonomic and functional profiles. Bioinformatics. 2014;30(21):3123–4. 10.1093/bioinformatics/btu494 25061070 PMC4609014

[41] RStudioTeam. RStudio: Integrated Development for R. RStudio, Inc., Boston, MA URL http://www.rstudio.com/. 2015.

[42] Pérez-Losada M, Crandall KA, Freishtat RJ. Comparison of two commercial DNA extraction kits for the analysis of nasopharyngeal bacterial communities. AIMS Microbiology. 2016;2(2):108–19.

[43] Huang YJ, Nelson CE, Brodie EL, Desantis TZ, Baek MS, Liu J, et al Airway microbiota and bronchial hyperresponsiveness in patients with suboptimally controlled asthma. The Journal of allergy and clinical immunology. 2011;127(2):372–81 e1–3. 10.1016/j.jaci.2010.10.048 21194740 PMC3037020

[44] Cardenas PA, Cooper PJ, Cox MJ, Chico M, Arias C, Moffatt MF, et al Upper airways microbiota in antibiotic-naive wheezing and healthy infants from the tropics of rural Ecuador. PloS one. 2012;7(10):e46803 10.1371/journal.pone.0046803 23071640 PMC3465279

[45] Yatsunenko T, Rey FE, Manary MJ, Trehan I, Dominguez-Bello MG, Contreras M, et al Human gut microbiome viewed across age and geography. Nature. 2012;486(7402):222–+. 10.1038/nature11053 22699611 PMC3376388

[46] Backhed F, Fraser CM, Ringel Y, Sanders ME, Sartor RB, Sherman PM, et al Defining a Healthy Human Gut Microbiome: Current Concepts, Future Directions, and Clinical Applications. Cell Host & Microbe. 2012;12(5):611–22. 23159051 10.1016/j.chom.2012.10.012

[47] Shade A, Handelsman J. Beyond the Venn diagram: the hunt for a core microbiome. Environ Microbiol. 2012;14(1):4–12. 10.1111/j.1462-2920.2011.02585.x 22004523

[48] Shafquat A, Joice R, Simmons SL, Huttenhower C. Functional and phylogenetic assembly of microbial communities in the human microbiome. Trends Microbiol. 2014;22(5):261–6. 10.1016/j.tim.2014.01.011 24618403 PMC4008634

[49] Biesbroek G, Wang X, Keijser BJ, Eijkemans RM, Trzcinski K, Rots NY, et al Seven-valent pneumococcal conjugate vaccine and nasopharyngeal microbiota in healthy children. Emerging infectious diseases. 2014;20(2):201–10. 10.3201/eid2002.131220 24447437 PMC3901477

[50] Pérez-Losada M, Crandall KA, Freishtat RJ. Two sampling methods yield distinct microbial signatures in the nasopharynx of asthmatic children. Microbiome. 2016 (in press). 10.1186/s40168-016-0170-5 PMC4910261 27306800

