# Supplementary figures and images for: Nasopharyngeal Microbiome Diversity Changes over Time in Children with Asthma
Source: PLoS One. 2017 Jan 20;12(1):e0170543. doi: 10.1371/journal.pone.0170543 (PMC5249091; doi:10.1371/journal.pone.0170543)

Proportion of sequences

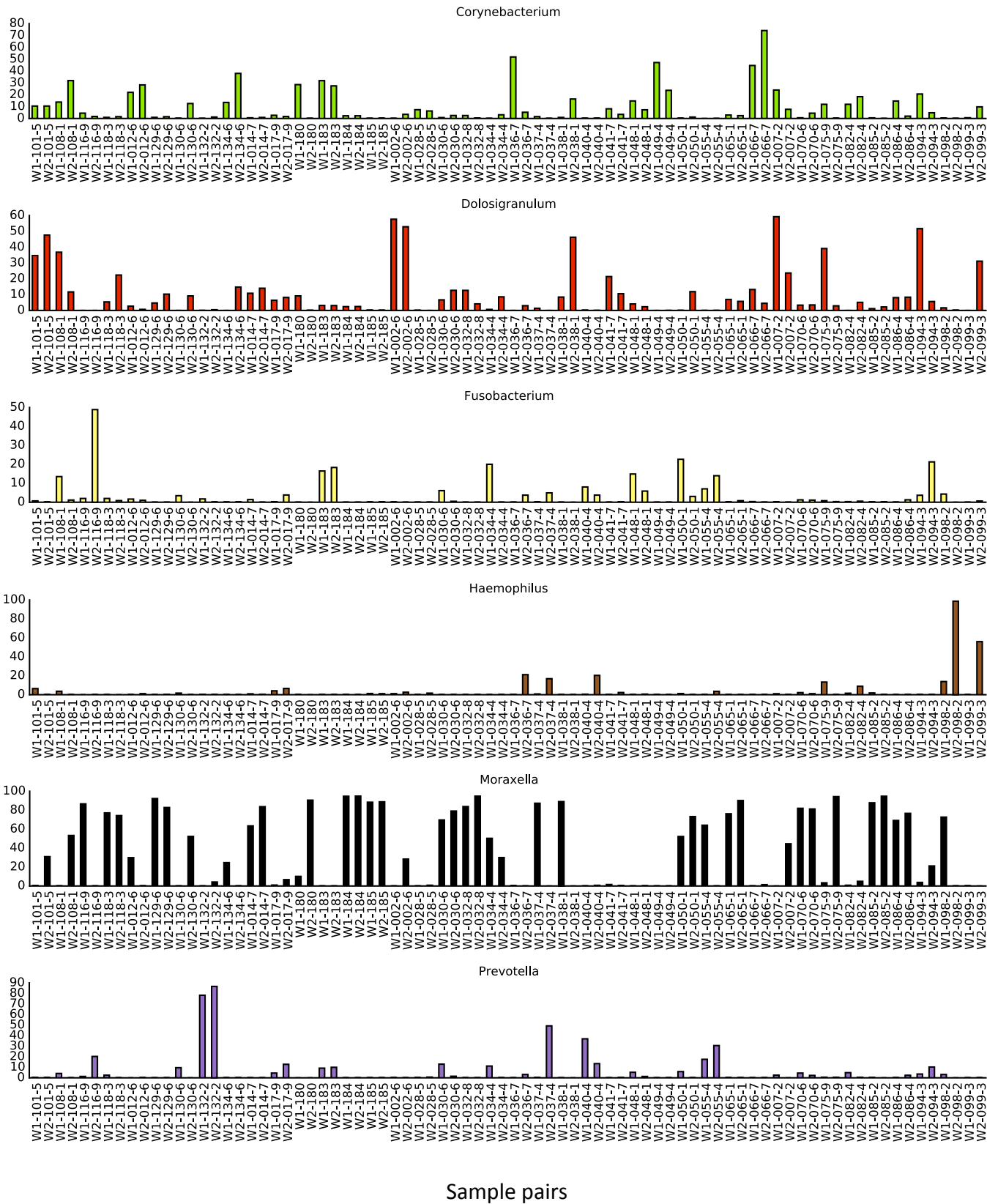

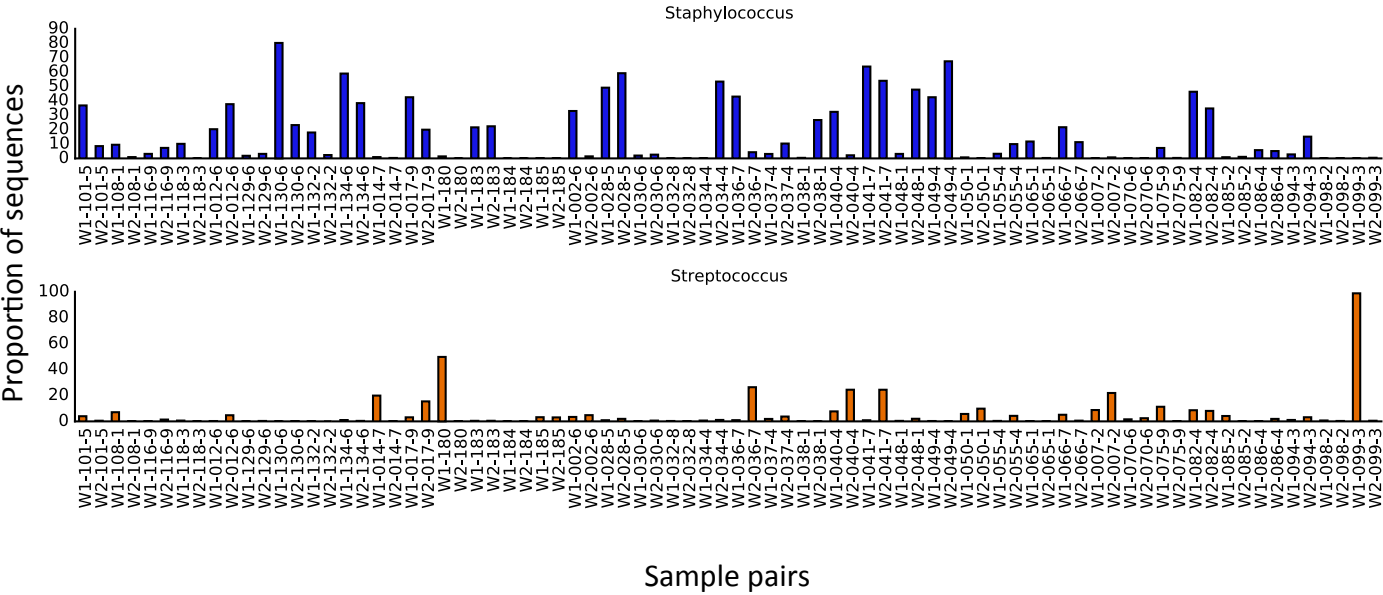

Supplement: S1 Fig — (PDF) [file pone.0170543.s004.pdf]
